# Clozapine is responsible for the *in vivo* imaging and pharmacological effects of clozapine-N-oxide in murine DREADD models

**DOI:** 10.3389/fphar.2025.1671065

**Published:** 2025-09-25

**Authors:** Jian Wang, Jiale Qin, Katsushi Kumata, Ming-Rong Zhang, Jun Qian, Wen Zhou, Bin Ji

**Affiliations:** ^1^ Department of Molecular Imaging and Nuclear Medicine, National Clinical Research Center for Cancer, Tianjin Medical University Cancer Institute and Hospital, Tianjin, China; ^2^ Tianjin’s Clinical Research Center for Cancer, Tianjin, China; ^3^ Key Laboratory of Cancer Immunology and Biotherapy, Ministry of Education, Tianjin Medical University, Tianjin, China; ^4^ Department of Radiopharmacy and Molecular Imaging, Minhang Hospital and School of Pharmaceutical Sciences, Fudan University, Shanghai, China; ^5^ Department of Advanced Nuclear Medicine Sciences, Institute for Quantum Medical Science, National Institutes for Quantum Science and Technology, Chiba, Japan; ^6^ Key Laboratory of Smart Drug Delivery (Fudan University), Ministry of Education, Fudan University, Shanghai, China; ^7^ State Key Laboratory of Advanced Drug Formulations for Overcoming Delivery Barriers, Shanghai, China; ^8^ Tianjin Key Laboratory of Technologies Enabling Development of Clinical Therapeutics and Diagnostics, School of Pharmacy, Tianjin Medical University, Tianjin, China; ^9^ Institute for Small-Molecule Drug Discovery & Development, Quzhou Fudan Institute, Quzhou, China

**Keywords:** designer receptors exclusively activated by designer drugs, human M4 mAChR DREADDs, *ex vivo* autoradiography, clozapine, clozapine-N-oxide

## Abstract

Although clozapine-N-oxide (CNO), a major metabolite of clozapine (CLZ), is a widely used agonist of designer receptors exclusively activated by designer drugs (DREADDs), the compound responsible for visualizing or activating DREADDs in nuclear medicine imaging and at pharmacological doses in murine models remains unclear. In this study, we performed positron emission tomography (PET) imaging and *ex vivo* autoradiography with ^11^C-CNO and ^11^C-clozapine (^11^C-CLZ) to detect human M4 muscarinic acetylcholine receptor DREADD (hM4D) expression in both a viral vector-injected intracranial mouse model and an original transgenic (Tg) mouse line. PET and autoradiographic images confirmed that both ^11^C-CNO and ^11^C-CLZ enabled visualization of hM4D expression in the brain. However, metabolite analysis revealed that the brain concentration of ^11^C-CLZ was approximately 40 times higher than that of ^11^C-CNO, while its plasma concentration was only 40% of that of ^11^C-CNO at 60 min post-injection. In both Tg and non-Tg mice intraperitoneally administered with a pharmacological dose of CNO (1 mg/kg), the ratios of non-radiolabeled CLZ to CNO ranged from 25 to 263 in the brain, whereas the ratios ranged from 0.04 to 0.11 at 30 min and 60 min post-injection in the plasma. Notably, the intraperitoneal administration of a low CLZ dose (0.1 mg/kg) induced a robust neuronal silencing effect exclusively in hM4D Tg mice. These findings clearly demonstrate that CLZ, not CNO, is the primary contributor to *in vivo* imaging signals and pharmacological effects in murine DREADD models. Additionally, our study confirms that the original hM4D Tg mouse line is a suitable model with stable DREADD expression for developing novel DREADD agonists.

## Introduction

Chemogenetic manipulation of neuronal activities with designer receptors exclusively activated by designer drugs (DREADDs) is a useful approach that can potentially be used for reversible and remote control of the neuronal activity of DREADD-expressing cells. Mutations in two conserved orthosteric-site residues of the human M4 muscarinic acetylcholine receptor (mAChR) result in a loss of responsiveness to acetylcholine and gain of reactivity with the synthetic ligand clozapine-N-oxide (CNO), a major metabolite of clozapine (CLZ) (Alexander et al., 2009; Armbruster et al., 2007). CNO should stimulate G_i_-coupled human M4 mAChR DREADDs (hM4D) and G_q_-coupled human M3 mAChR DREADDs (hM3D) exclusively, resulting in neuronal silencing (hM4D) or ongoing firing (hM3D), respectively, without affecting DREADD-negative cells (Roth, 2016). DREADDs have been reported to modify neuronal function, enabling on-demand behavioral control in both rodents and nonhuman primates (Alexander et al., 2009; Nagai et al., 2016; Eldridge et al., 2016). Chemogenetic approaches have also been used to ameliorate symptoms of neurological and neuropsychiatric disorders (Fortress et al., 2015; Kätzel et al., 2014; Dell'Anno et al., 2014) in animal models, indicating considerable potential for future human therapeutics. Therapeutic DREADD expression is often achieved by direct intracranial injection of a viral vector (a locally infectious but non-replicating virus).

Longitudinal, noninvasive monitoring of therapeutic DREADD expression in the targeted tissue element (neuron or glia) would provide valuable tempospatial information about therapeutic gene expression. Positron emission tomography (PET) enables the high-sensitivity mapping of target molecules with the use of a specific radioligand and offers an imaging-based, quantifiable biomarker. We demonstrated the utility of PET with ^11^C-labeled CNO (^11^C-CNO) for detecting hM4D expression in our originally developed transgenic (Tg) mouse line (Ji et al., 2016). Although Nagai et al. (2020) showed no overt conversion from CNO to CLZ in nonhuman primates, recent experimental evidence indicates that converted CLZ readily enters the brain, occupies central nervous system-expressed DREADDs, and induces preferential DREADD-mediated behaviors in mouse and rat models (Jendryka et al., 2019; Manvich et al., 2018; Gomez et al., 2017), indicating a species difference between rodents and nonhuman primates. Given that murine models are the most widely used experimental animals in neuroscience studies, it is very important to clarify the real substance responsible for the noninvasive visualization or functional activation of DREADDs in ^11^C-CNO-PET or CNO-modified neuronal action in nuclear medicine imaging and in murine models. Jendryka et al. (2019) found that after systemic administration of high-dose CNO (3.5 mg/kg) in normal mice, free CNO levels in the CSF and the total brain concentration were both higher than the median effect concentration (EC_50_) at hM4D and hM3D during the early phase. However, such a high dose of CNO (3.5 mg/kg) is likely to activate off-target effects due to the high amount of converted CLZ (Manvich et al., 2018). Additionally, the ratio of converted CLZ differed significantly in the presence versus absence of DREADDs in the rat brain (Gomez et al., 2017), raising the question of whether the results from normal brains accurately reflect the situation in the mouse brain with DREADD expression. Thus, it is essential to clarify the actual compound responsible for nuclear medicine imaging and the pharmacological effects.

In the present study, *ex vivo* autoradiography with ^11^C-CNO and ^11^C-CLZ, along with metabolite analysis, was conducted using our original hM4D transgenic (Tg) mouse line at nuclear medicine imaging and pharmacological dose levels of CNO to identify the component responsible for *in vivo* imaging and pharmacological effects in the murine model. We investigated the utility of the hM4D Tg mouse line as a tool for sensitive and stable evaluation of the pharmacological properties of DREADD agonists.

## Materials and methods

### Ethics statement

The mice studied here were maintained and handled in accordance with the National Research Council’s Guide for the Care and Use of Laboratory Animals and the guidelines of the corresponding affiliation. The protocols for these animal experiments were approved by the Animal Ethics Committees of Fudan University and the Institute for Quantum Medical Science, National Institutes for Quantum and Science and Technology. Experiments were performed and reported in accordance with the ARRIVE (Animal Research: Reporting of *In Vivo* Experiments) guidelines.

### Animal models

A high-expressing transgenic line containing 29 copies of the hM4D transgene, as previously reported (Ji et al., 2016), was used in the present study. The mice were kept and handled in accordance with the National Research Council’s Guide for the Care and Use of Laboratory Animals and our institutional guidelines. Mice heterozygous for the transgene, along with their non-Tg littermates, were used for further studies.

### Reagents and antibodies

The following reagents were of analytical grade and were commercially purchased: CLZ and CNO were purchased from Enzo Life Sciences, Inc. (Farmingdale, NY, United States). The following commercial antibodies were employed in this study: rabbit polyclonal antibodies against M4 mAChR (H-175; Santa Cruz Biotechnology Inc., Santa Cruz, CA, United States) and GFP (Living Colors^®^; Clontech Laboratories Inc., Mountain View, CA, United States). All other chemicals were of analytical grade and were purchased commercially.

### Intracranial injection of lentivirus into the adult mouse brain

A lentiviral vector expressing an hM4Di-cyan fluorescent protein (CFP) fusion protein under a neuron-specific human synapsin promoter (lenti-hM4Di-CFP vector) was generated as previously described (Eldridge et al., 2016). Intracranial injection of the lentiviral vector into the brains of WT mice was conducted as previously described (Ji et al., 2008). Briefly, mice were anesthetized with 1.5% (v/v) isoflurane and placed in a stereotactic frame (Narishige, Tokyo, Japan). Using a 10-µL Hamilton syringe, 1 µL of lentivirus solution was injected over 2 min into the right hippocampus (stereotactic coordinates: anteroposterior, 2.8 mm; mediolateral, 2.0 mm; dorsoventral, 2.0 mm) unless otherwise indicated. The needle was left in place for 3 min before being withdrawn. These mice were scanned with a microPET system at 21 days post-injection.

### Magnetic resonance imaging (MRI) of mouse brains

MRI scans were performed 20 days after viral vector injection as follows. The mice were anesthetized with 1.5% (v/v) isoflurane and secured using ear bars and a rigid facemask during the MRI scans. T2-weighted 2D multi-slice spin-echo (rapid acquisition with relaxation enhancement; RARE) was performed using a 7.0-Tesla MRI system (Bruker BioSpin, AVANCE-III) equipped with a volume coil for transmission (Bruker BioSpin) and a surface coil for reception (RAPID Biomedical, Germany) by the following parameters: repetition time (TR) = 4,200 ms; effective echo time (TE) = 36 ms; field of view (FOV) = 25.6 × 14.5 mm^2^; slice thickness = 0.5 mm; number of slices = 28 (no gap); matrix = 256 × 256; number of acquisitions (NA) = 8; nominal in-plane resolution = 100 × 57 μm^2^; and RARE factor = 8.

### Radiosynthesis and small animal PET imaging

^11^C-CNO and ^11^C-CLZ were radiosynthesized by reacting N-desmethylclozapine with [^11^C]CH_3_I as described in a previous report (Bender et al., 1994). Their radiochemical purity and specific radioactivity exceeded 97% and 37 GBq/μmol, respectively, at the end of synthesis. PET scans were performed using a microPET Focus 220 animal scanner (Siemens Medical Solutions USA, Knoxville, TN) as described elsewhere (Bender et al., 1994). Emission scans were acquired for 60 min in a 3D list mode with an energy window of 350–750 keV immediately after ^11^C-CNO (55–68 MBq) was intravenously injected. Images were reconstructed using either the maximum a posteriori method or filtered back-projection using a 0.5-mm Hanning filter. Volumes of interest (VOIs) were placed on lentivirus-injected sites and contralateral counterparts of WT mice, using PMOD^®^ image analysis software (PMOD Technologies Ltd., Zurich, Switzerland), with corresponding MRI-T2 images of each individual mouse. *In vivo* binding of ^11^C-CNO was determined as [(target-to-cerebellum ratio of radioactivity (average of data at 30–60 min) – 1] (Ji et al., 2016).

After the PET scans, the mice were deeply anesthetized with sodium pentobarbital and were transcardially perfused with phosphate-buffered saline. The brain tissues were removed and fixed with 4% paraformaldehyde in phosphate buffer (PB) overnight, followed by cryoprotection with 30% sucrose in PB for brain section preparation.

### Immunohistochemical and histochemical analyses

Frozen sections, 10-μm-thick, were generated in a cryostat (HM560; Carl Zeiss, Jena, Germany), and subsequently immunostained using fluorophore-conjugated secondary antibodies (Molecular Probes/Invitrogen, Eugene, OR) as described elsewhere (Ji et al., 2013). Similarly, immunofluorescence staining of cultured cells on a cover glass was carried out. All stained samples were examined by an all-in-one fluorescence microscope (BZ-9000; Keyence, Osaka, Japan) that could tile photomicrographs and merge them into a high-resolution image with a large field of view.

### *Ex vivo* autoradiographic analysis

Tg mice and age-matched non-Tg mice received a bolus injection of radioligand (37 MBq of ^11^C-CNO or ^11^C-CLZ) via the tail vein. The animals were deeply anesthetized with isoflurane and euthanized by decapitation 45 min after injection. The brains were immediately removed without perfusion, frozen in dry ice, and sectioned into 20-µm-thick slices using a cryostat microtome (Carl Zeiss, Oberkochen, Germany). These brain sections were then placed in close contact with an imaging plate for an optimized exposure time and scanned with the BAS-5000 system. Regions of interest (ROIs) were carefully placed on the indicated brain regions based on a standard brain atlas (The Mouse Brain in Stereotaxic Coordinates, Paxinos G and Franklin KBJ, Second Edition, Academic Press, San Diego, 2001).

#### Locomotor activity test

A locomotor activity test was performed from 1:00 p.m. to 5:00 p.m. (light cycle). hM4D Tg and non-Tg mice were intraperitoneally injected with 0.1 mg/kg of CLZ or vehicle and were allowed to move freely in a home cage that was equipped with a computer-operated animal activity monitoring system (Ohara Ika Sangyo, Tokyo, Japan). Data collection over 150 min was initiated immediately after the CLZ administration. The total distance moved was measured as an index of locomotor activity.

### Radioactive metabolite analysis

The hM4D Tg and non-Tg mice (12 weeks old, 24.3 ± 1.42 g) received intravenous injections via the tail vein with ^11^C-CNO (37 MBq) and were subsequently euthanized by cervical dislocation at 15 min, 30 min, and 60 min (n = 1 for each time point) after the injection. Blood and brain samples were obtained quickly and treated according to a previously reported procedure (Zhang et al., 2007), with slight modifications. An aliquot of the supernatant (0.2–1.0 mL) obtained from the plasma or brain homogenate was injected into a radioactivity-detecting HPLC system and analyzed using a YMC PackPro C18 column (4.6 mm i.d. × 150 mm) with a mobile phase of CH_3_CN/H_2_O (Et3N 0.1%) (60/40) at a flow rate of 1.0 mL/min. HPLC retention times of ^11^C-CNO and ^11^C-CLZ were identified by non-radioactive standards of CNO and CLZ. The percentage ratio of ^11^C-CLZ to ^11^C-CNO on the HPLC chromatogram was calculated as % = (peak area for ^11^C-CLZ/^11^C-CNO) × 100.

### LC/MS/MS measurement

After an intraperitoneal (i.p.) injection of CNO (1 mg/kg), blood and brain samples were collected at 15 min, 30 min, or 60 min. Mouse blood was collected from the heart using heparinized syringes under isoflurane anesthesia and centrifuged at 10,000 g for 5 min to obtain plasma samples, and brains were removed and snap-frozen in liquid nitrogen following blood collection. All samples were stored at −80 °C until analysis. The protocol for sample pretreatment of plasma has been previously described (Nagai et al., 2016). Briefly, samples (50 µL) were diluted in 150 µL of methanol, followed by the addition of a further 50 µL of 50% methanol and 20 µL of granisetron solution (10 ng/mL, internal standard). The diluted samples were vortexed briefly, then ultracentrifuged at 10,600 g at 4 °C for 2 min. A 50 μL aliquot of supernatant from each sample was filtered and diluted to 1:10 or 1:300 with water. Mouse brains were homogenized in a two-fold volume of water. An acetonitrile solution (0.8 mL) containing granisetron (0.5 ng/mL) as an internal standard was added to the brain homogenate (0.2 mL), followed by centrifugation at 10,600 × g for 2 min at 4 °C. The supernatant was collected and filtered through a solid-phase extraction column (Phree, Shimadzu). The filtrate was dried under nitrogen gas at 40 °C and re-dissolved in 5% acetonitrile (0.2 mL), followed by sonication for 30 s and centrifugation (500 g) for 2 min. The supernatant was filtered through a solid-phase extraction column again before its application to LC-MS/MS.

Quantification of CNO and CLZ was performed by multiple reaction monitoring (MRM) using a Shimadzu UHPLC LC-30AD system coupled with an AB Sciex QTRAP 6500 tandem mass spectrometer. A Capcell Pak C18 MGIII column (Shiseido; 5 μm, 2.0 mm × 50 mm) was used for metabolite separation. UHPLC was conducted using the following mobile phases: mobile phase A, 0.1% formic acid in water; mobile phase B, 0.1% formic acid in acetonitrile. A linear gradient was applied, starting from 5% B and increasing to 20% at 2.5 mins, followed by an increase to 95% at 3 mins, which was held briefly before returning to the initial condition and equilibrating for an additional 2 min. The flow rate was 0.4 mL min^−1^, and the column temperature was maintained at 40 °C. The AB Sciex QTRAP 6500 system was operated in MRM ESI + mode. The following MRM transitions (Q1/Q3) were used to monitor each compound: CLZ (327.1/270.0), CNO (343.1/192.1), and granisetron (313.2/138.1). The internal standard-normalized area under the peak (response) from the serially diluted authentic standard solution was used to construct a calibration curve for each compound. The concentration of compounds was calculated by multiplying the calibration curve value by any dilution factor.

### Statistical analysis

Statistical analyses in the present study were performed by SPSS software (Chicago, IL). For comparisons between groups, we performed two-way analysis of variance (ANOVA) or a Student’s t-test as indicated in the figure legends. The difference between groups was considered significant when the p-value was less than 0.05.

## Results

### ^11^C-CNO-PET enabled visualization of viral vector-mediated hM4D expression in the living brain

**FIGURE 1:**
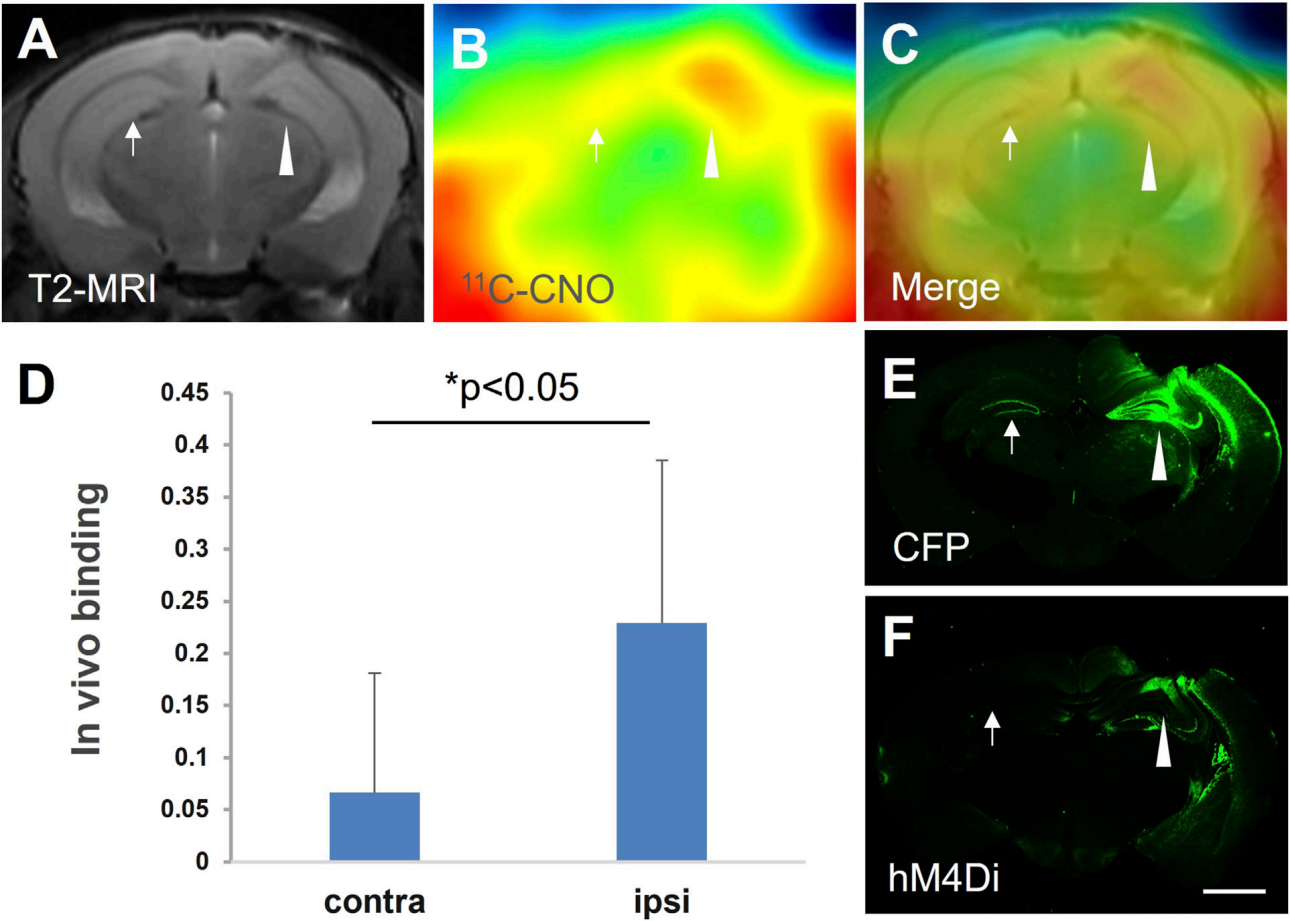
hM4D expression mediated by intracranial injection of the lenti-hM4Di-CFP vector captured by ^11^C-CNO-PET and *in vitro postmortem* analysis. Representative MRI T2 **(A)** and ^11^C-CNO-PET **(B)** images of coronal mouse brain sections containing the hippocampus at 21 days after intrahippocampal injection of the vector. The PET image of ^11^C-CNO was generated from averaged dynamic data (30–60 mins), and a merged image of the MRI and PET images is shown in panel **(C)**. *In vivo* binding of ^11^C-CNO showed significantly greater specific radioactivity retention in the ipsilateral (ipsi) hippocampus than that in the contralateral (contra) corresponding area (n = 3, t = −3.18, *, p < 0.05, by paired t-test) **(D)**. Based on postmortem immunohistochemical images of hM4D **(F)** and CFP **(E)**, the brain region with increased radioactivity retention was very spatially consistent with hM4D and CFP expression (arrowheads and arrows indicate the vector injection site and the contralateral area, respectively, in panels **(A–C,E,F)**. Error bars represent standard deviation (SD). Scale bars: 1 mm **(E,F)**.

Under stereotaxic guidance, three mice were injected with a lentiviral vector encoding hM4Di fused to cyan fluorescent protein (CFP) into the right hippocampus. The animals were scanned with ^11^C-CNO after 21 days. A representative PET image showed the focal ^11^C-CNO uptake on the viral-injected side. The PET image, co-registered with MRI, demonstrated that the focal uptake was localized to the presumed hM4D expression area around the viral injection sites (Figures 1A–C). *In vivo* binding was quantified using the cerebellum as the reference region, and a significant increase in radioligand retention was observed in the ipsilateral hippocampus compared to the contralateral side (Figure 1D). Postmortem immunohistochemical analysis using an anti-hM4 antibody demonstrated that the focal PET signal corresponded to hM4D expression (Figure 1F), with co-localized expression of the fusion protein CFP also detected (Figure 1E).

### *Ex vivo* autoradiography with ^11^C-CNO and ^11^C-CLZ in hM4D Tg mouse

**FIGURE 2:**
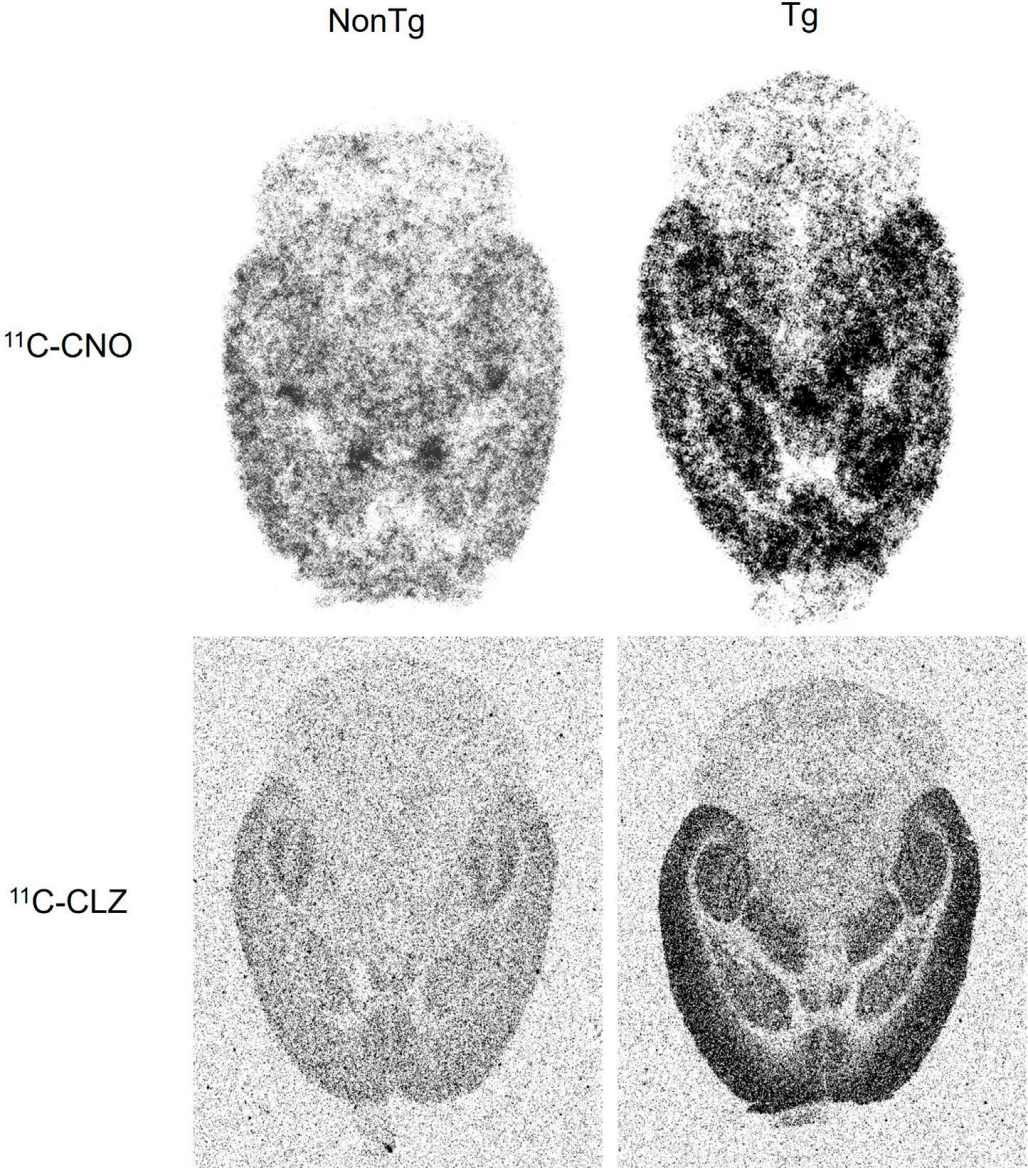
*Ex vivo* autoradiographic images of ^11^C-CNO and ^11^C-CLZ in hM4D Tg and non-Tg mouse brains. Horizontal brain images from Tg (right) and age-matched non-Tg (left) mice, which received a bolus injection of either ^11^C-CNO (upper panel) or ^11^C-CLZ (lower panel).

*Ex vivo* autoradiography demonstrated a similar accumulation pattern of ^11^C-CNO and ^11^C-CLZ, with higher uptake observed in the forebrain of the Tg mice than in the non-Tg mice, and no apparent difference observed in cerebellar uptake between the two groups (Figure 2). These findings were consistent with ^11^C-CNO- and ^11^C-CLZ-PET images, as previously reported (Ji et al., 2016). When combined with hematoxylin and eosin (HE) staining, the autoradiographic images provided superior anatomical resolution than PET, enabling the identification of abundant hM4D expression and the specific binding of the radioactive component in the cortex, hippocampus, anterior pretectal nucleus, and striatum in the Tg mouse brain (Supplementary Figure S1).

### Radioactive metabolites of ^11^C-CNO in the brain and plasma

**FIGURE 3:**
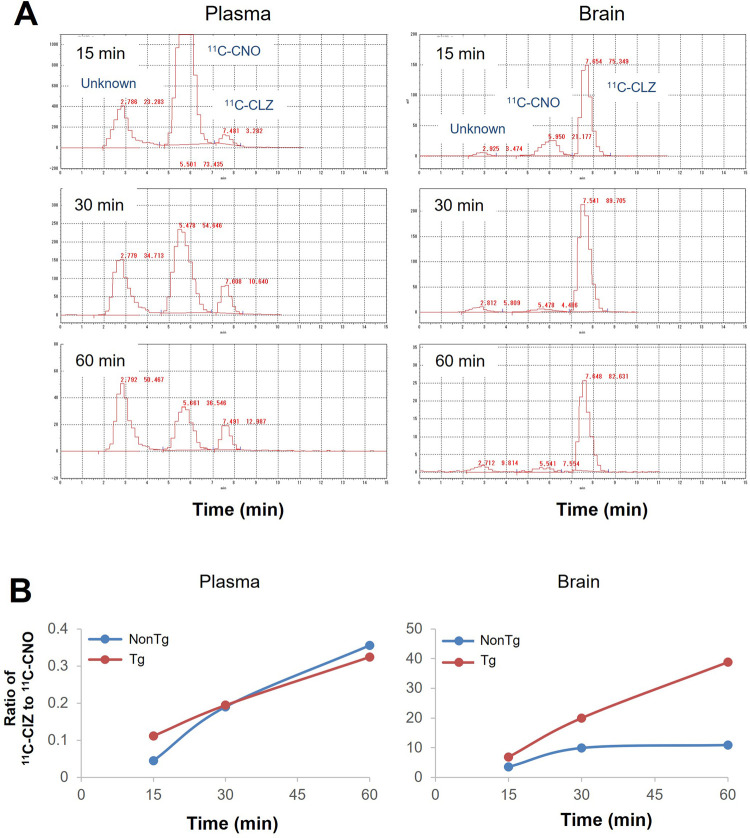
Radioactive metabolites of ^11^C-CNO in the brain and plasma samples. **(A)** HPLC charts for the analysis of radioactive metabolites in the forebrain (lower panel) and plasma (upper panel) of an hM4D Tg mouse after i.v. injection of ^11^C-CNO. The samples were collected after 15 min, 30 min, and 60 min. Retention times for HPLC were 2.7 min for an unknown metabolite, 5.5 min for ^11^C-CNO, and 7.5 min for ^11^C-CLZ. **(B)** Quantitative analysis of ^11^C-CNO and ^11^C-CLZ in the brain (lower panel) and plasma (upper panel) samples from hM4D Tg and non-Tg mice at the different time points.

The HPLC system equipped with a radiation detector identified three radioactive components, including the parent compound and two metabolites, ^11^C-CLZ and an unknown component, in plasma and brain samples from mice that received a bolus systemic injection of ^11^C-CNO. ^11^C-CLZ was a major component in the brain at all time points (Figure 3A). The ratios of ^11^C-CLZ/^11^C-CNO continuously increased in the plasma and did not show overt differences between the Tg and non-Tg mice. In contrast, the ratios were overtly higher in Tg mouse brains than in the brains of non-Tg mice after 30 min (Figure 3B).

### Metabolite analysis for the mice receiving a pharmacological dose of CNO

Metabolite analysis with LC/MS/MS also confirmed the rapid conversion of CNO to CLZ at the pharmacological dose of CNO. Like the case of ^11^C-CNO, the major component in the brains was CLZ at all indicated time points in both Tg and non-Tg mice, with concentrations at least 25 times higher than CNO. Given that the CLZ/CNO ratios were approximately 10% in the plasma, CNO detected in brain samples was likely due to blood contamination. Despite the higher retention of ^11^C-CLZ in Tg mouse brains, there was no significant difference in the CLZ/CNO ratio between Tg and non-Tg mouse brains (Table 1).

**TABLE 1:** Amounts of CNO and CLZ in the brain and plasma samples of hM4D Tg and non Tg mice after being administered a pharmacological dose of CNO.

	Concentration in the brain (ng/g tissue)				Concentration in plasma (ng/mL)			
	30 min		60 min		30 min		60 min	
	Tg	Non-Tg	Tg	Non-Tg	Tg	Non-Tg	Tg	Non-Tg
CNO	1.00 ± 0.3	1.00 ± 0.4	0.40 ± 0.4	0.10 ± 0.3	210 ± 50.9	180 ± 42.6	60.2 ± 25.3	54.8 ± 36.0
CLZ	26.9 ± 3.9	51.0 ± 19.2	29.1 ± 2.2	39.3 ± 4.2	7.5 ± 1.6	11.8 ± 1.4	5.8 ± 0.7	6.2 ± 2.1
CLZ/CNO	25.7	49.7	65.5	263	0.04	0.07	0.10	0.11

The samples were collected from hM4D Tg and non-Tg mice at 30 and 60 min after i.p. administration of CNO (1 mg/kg). N = 4 for each group.

### Locomotive activity test in hM4D Tg mice receiving a low dose of CLZ

**FIGURE 4:**
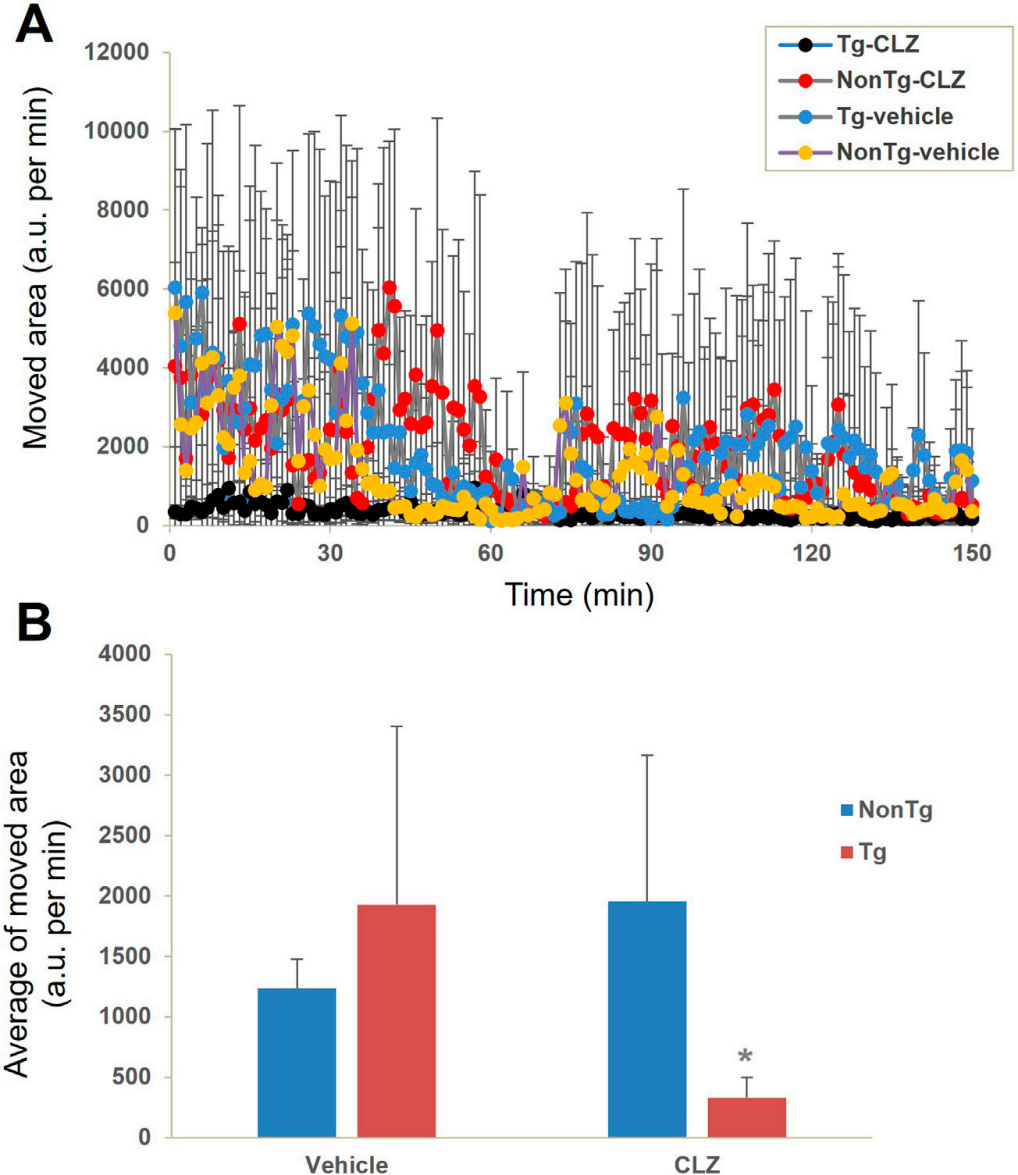
Neuronal silencing exclusively induced by CLZ in hM4Di Tg mice. Locomotor activity was evaluated by measuring the change in the area occupied by non-Tg and hM4D Tg mice intraperitoneally administered with the vehicle or CLZ (0.1 mg/kg) prior to assessment (n = 4 per group). **(A)** Time course of movement indexed by the change in the location of the mouse. **(B)** Average of movement from 0 to 150 min. There was a significant main effect of interaction between CLZ administration and genotype [F (1, 12) = 5.764, p < 0.05 by two-way ANOVA] but no significant main effects were observed for CLZ administration [F (1, 12) = 0.929, p > 0.05 by two-way ANOVA] or animal genotype [F (1, 12) = 0.829, p > 0.05 by two-way ANOVA]. *Post hoc* analysis revealed that there was a significant decrease in locomotor activity in CLZ-treated Tg mice compared to vehicle-treated Tg or CLZ-treated non-Tg mice (*, p < 0.05, Mann–Whitney U test), but no significant difference was observed between vehicle- and CLZ-treated non-Tg mice (p > 0.05, Mann–Whitney U test).

We then assessed the capability of a low dose of CLZ to control DREADD-mediated neuronal activity without stimulating the endogenous mAChR signaling pathway. Non-Tg and hM4D Tg mice with vehicle administration presented similar locomotor activity in an open field, while an intraperitoneal injection of 0.1 mg/kg of CLZ induced immobilization of the hM4D Tg mice, in contrast to no behavioral alterations in the non-Tg mice (Figure 4).

## Discussion

The present data provide compelling evidence that ^11^C-CNO imaging-based quantitative assessment of intracranial lentivirus vector injection-mediated or transgenic hM4D expression and CNO-induced neuronal silencing in hM4D Tg mice (Nagai et al., 2020) is due to the converted ^11^C-CLZ binding to DREADDs. There are large species differences between rodents and nonhuman primates. Gomez et al. demonstrated that CNO is metabolized into CLZ, which is the key molecule for the activation of DREADDs in a rat DREADD model (Gomez et al., 2017), while Nagai et al. claimed that little CNO can be converted to CLZ in nonhuman primates (Nagai et al., 2016). The present data showed that CLZ concentration was at least 20 times higher than that of CNO at either nuclear medicine imaging or pharmacological doses in either hM4D Tg or non-Tg mouse brains (Table 1; Figure 3). Given that the CLZ-to-CNO ratio is not higher than 1 even in the DREADD rats, while the major component is still CNO in the brains of the control rats (Gomez et al., 2017), the difference in the CNO-to-CLZ conversion is great between mice and rats. This may explain why ^11^C-CNO-PET can noninvasively detect DREADD expression in the mouse brain (Figure 1) but not in the rat brain (Gomez et al., 2017), despite no overt difference in viral vector-mediated DREADD expression level between these animals. Based on the above findings in present and previous studies, we conclude that clozapine is the key molecule for DREADD imaging and DREADD-mediated neuronal activity modification in murine models.

Until now, CNO has been one of the most widely used DREADD agonists. There are 22 peer-reviewed publications in the PubMed database containing the keywords DREADD and CNO that were published in 2024. The present findings strongly suggest the use of alternative DREADD agonists to replace CNO to avoid the influence of individual or animal species differences in the conversion of CNO to CLZ. Apart from CNO, there are several alternative DREADD agonists, including DCZ (Nagai et al., 2020), C21 (Thompson et al., 2018), and JHU37160 (J60) (Bonaventura et al., 2019). Among them, DCZ showed high affinity and selectivity for DREADDs as both imaging agent and pharmacological agonists (Nagai et al., 2020). Five articles were found in the PubMed database using the keywords “DREADD” and “DCZ” in 2024, next only by 22 hits mentioning CNO. The present study showed that the i.p. administration of CLZ with a low dose (0.1 mg/kg) rapidly and dramatically suppressed the locomotor activity in hM4D Tg mice, while no significant pharmacological effects were noted in non-Tg mice (Figure 4). This dose is similar to the common dose of DCZ used in nonhuman primates (Nagai et al., 2020), indicating the excellent efficacy of clozapine as a DREADD agonist. Although DCZ showed better selectivity in human and nonhuman primates than CLZ based on PET imaging and the PRESTO-Tango assay (Nagai et al., 2020), it might not be an advantage in a murine model. Our unpublished data showed no significant difference in the selectivity or sensitivity of the noninvasive detection of transgenic hM4D expression between ^11^C-CLZ and ^11^C-DCZ (data not shown). Recently, Zhang et al. (2022) reported four DREADD-related cryogenic electron microscopy high-resolution structures: an hM3D-miniGq complex and an hM4D-miniGo complex bound to DCZ; an hM3D-miniGq complex bound to CNO; and an hM3R-miniGq complex bound to iperoxo. Thus, to clarify the potential of CLZ as a DREADD agonist, the next step should be to investigate the DREADD–G-protein complex bound to CLZ.

Unstable behavioral responses and DREADD expression levels greatly complicate the evaluation of the pharmacological effects of DREADD agonists. For example, several well-used DREADD agonists, CNO, C21, and DCZ, did not affect the locomotor activity in DREADD viral vector-infected animals, likely due to large individual differences in both behavioral responses and receptor expression among individuals (Robinson et al., 2023). Immunohistochemistry typically offers higher resolution than *ex vivo* autoradiography. However, the quality of immunostaining depends on antibody specificity. In this case, the used antibody H175 targets the human M4 receptor. While it enables immunostaining of hM4D in the mouse brain, it also detects endogenous mouse M4 receptors. As shown in our previous publication (Ji et al., 2016), immunohistochemistry revealed strong immunoreactivity in the striatum of both non-Tg and hM4D Tg mice, making it difficult to determine hM4D expression specifically in the Tg line. In contrast, *ex vivo* autoradiography with ^11^C-CNO and ^11^C-CLZ characterized the pattern of hM4D transgene expression in our original Tg mouse brain and identified the cortex, hippocampus, and striatum as high-level expression regions, while no detectable gene expression was observed in the cerebellum. Our present and previous data demonstrate a stable behavioral response when using our original hM4D Tg mouse line to measure the agonistic effect of CNO (Ji et al., 2016) and CLZ (Figure 4). With a small sample size of four mice for each group, we detected a statistically significant agonistic effect of CLZ in hM4D Tg mice, compared with the other three groups (Figure 4). These data indicate the superiority of our original hM4D Tg mouse line as a powerful tool for developing new effective DREADD agonists, offering a significant advantage in terms of stable DREADD expression levels, compared with virus vector-mediated DREADD expression levels with large individual differences.

In summary, in the present study, we have provided an hM4D Tg line for novel agonist development and identified CLZ as a critical component responsible for imaging and functional control of DREADDs in a murine model.

## Data Availability

The original contributions presented in the study are included in the article/Supplementary Material; further inquiries can be directed to the corresponding authors.
