# Supplementary material for: Clozapine is responsible for the in vivo imaging and pharmacological effects of clozapine-N-oxide in murine DREADD models
Source: Front Pharmacol. 2025 Sep 25;16:1671065. doi: 10.3389/fphar.2025.1671065 (PMC12507898; doi:10.3389/fphar.2025.1671065)
Supplement: Supplementary file 1 [file Supplementaryfile1.docx]

**Supplementary Material**

**Clozapine** **Responsible for** **In-vivo Imaging and Pharmacological Effect of Clozapine-N-oxide (CNO) in Murine DREADD Models**

**Supplementary Figures**

**
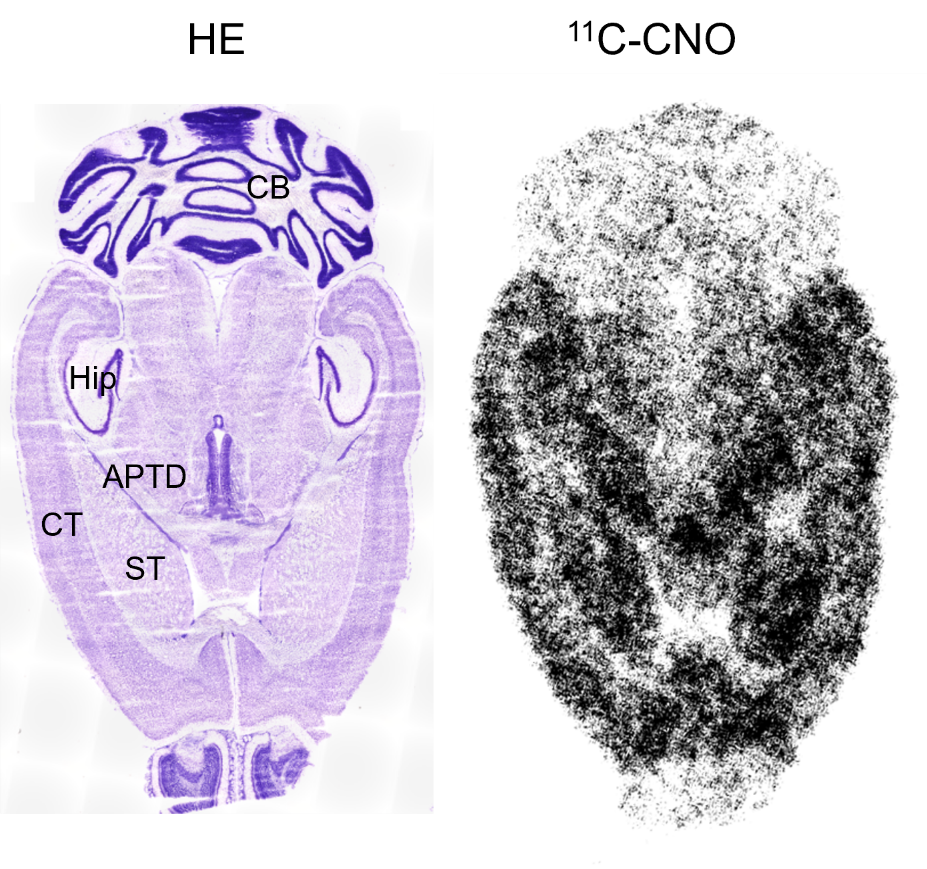
**

**Supplementary Figure 1.** Distribution of hM4D expression in Tg mouse. HE staining (left) and ex vivo autoradiography with ^11^C-CNO (right) in a same brain section from a hM4D Tg mouse clearly indicated brain regions with high radio-signals. CT: Cortex, ST: Striatum, Hip: Hippocampus, APTD: anterior pretectal nucleus, dorsal part, CB: Cerebellum
